# A geo-enabled digital tool for microplanning and delivery of indoor residual spray in Zambia: A case study, 2016–2020

**DOI:** 10.1371/journal.pgph.0004683

**Published:** 2025-11-20

**Authors:** Anne C. Martin, Frazer Bwalya, Christina Riley, Derek Pollard, Kentzo Mumba, Busiku Hamainza, Kafula Silumbe, David A. Larsen, Benjamin Winters, Brian Chirwa, Daniel J. Bridges, Anna Winters

**Affiliations:** 1 Department of Epidemiology, Johns Hopkins Bloomberg School of Public Health, Baltimore, Maryland, United States of America; 2 Akros Inc, Missoula, Montana, United States of America; 3 Akros Research, Lusaka, Zambia; 4 Zambia National Malaria Elimination Program, Lusaka, Zambia; 5 PATH, Macepa, Lusaka, Zambia; 6 Falk College of Sport and Human Dynamics, Syracuse University, Syracuse, New York, United States of America; 7 Abt Associates, Lusaka, Zambia; 8 School of Public and Community Health Science, University of Montana, Missoula, Montana, United States of America; London School of Hygiene & Tropical Medicine, UNITED KINGDOM OF GREAT BRITAIN AND NORTHERN IRELAND

## Abstract

Indoor residual spraying (IRS) is a vector control tool recommended by the WHO in areas of high malaria burden. Effective IRS implementation is complicated when up-to-date and accurate counts of structures eligible for IRS are unavailable; resultingly, programmatic spray coverage is typically calculated as the proportion of found structures that are sprayed. From 2016 – 2020 in Zambia, an open-source tool termed “Reveal,” was used to support digital mapping, microplanning, campaign monitoring and evaluation of IRS. Satellite imagery was used to enumerate structures and identify “spray areas,” clusters of structures for operational planning. Spray areas were visualized in the Reveal tool web-based planning module where campaign planners selected which would be sprayed and determined the resources required. Field teams used the Reveal tool mobile application to navigate and to record spray data against each structure. True coverage was calculated as the proportion of enumerated structures that were sprayed. Reveal was implemented in 21 districts across five years and coverage is reported for each district and year. Logistic regression models explored whether structures were more or less likely to be found and sprayed depending on a given calendar year’s targeting strategy, partner support level, and the year of Reveal implementation in a given district (first, second, third, or fourth). District-level programmatic IRS coverages overestimated the true adjusted coverages by an average 31.5 percentage points (range 2.8 - 69.4). The odds of finding and spraying structures increased with year of implementation; in the fourth year of implementation in a district, the odds of a household being sprayed and found were two times higher than in the first year of implementation. Digital tools improve structure and population estimates for planning and deployments in IRS campaigns, which may lead to more accurate coverage measurements and increased coverage over time.

## Background

Malaria vector control has seen substantial investment from national programs, global, multilateral, and bilateral partnerships. The President’s Malaria Initiative (PMI) partners with 27 countries and has cumulatively provided over $6.3 billion since 2005 [1]. The Global Fund for AIDS, Tuberculosis, and Malaria has invested over $12 billion in malaria control programs since 2002 [2]. Private entities, and countries themselves, also invest significantly in malaria control and prevention efforts. Indoor residual spray (IRS) and long-lasting insecticide treated nets (LLINs) are core vector control tools, and the median cost per person protected is notably higher for IRS ($6.70) than for LLINs ($2.20) [3]. The World Health Organization (WHO) recommends providing coverage with at least one of these core vector control tools in high-burden areas [1,4]. Such financial investments require precise programmatic measurement and adept program management. For IRS, WHO recommends reporting the number of rooms or structures sprayed (numerator) as a percentage of the total number of rooms or structures in areas targeted for IRS (denominator), and defines a successful IRS campaign as one that achieves 80% coverage [5]. As the largest global funder of IRS, PMI reports this same IRS coverage metric in the majority of the countries where it operates [6]. To inform accurate implementation planning and reporting, most IRS programs conduct detailed “microplanning” meetings, which involve selecting operational units where IRS will be deployed, then estimating total and targeted-for-spray structure counts within these units such that coverage will exceed the targeted coverage threshold [7]. Structure count estimates are usually derived from the last formal census or from aggregated community headcounts provided by health facilities.

**Fig 1:**
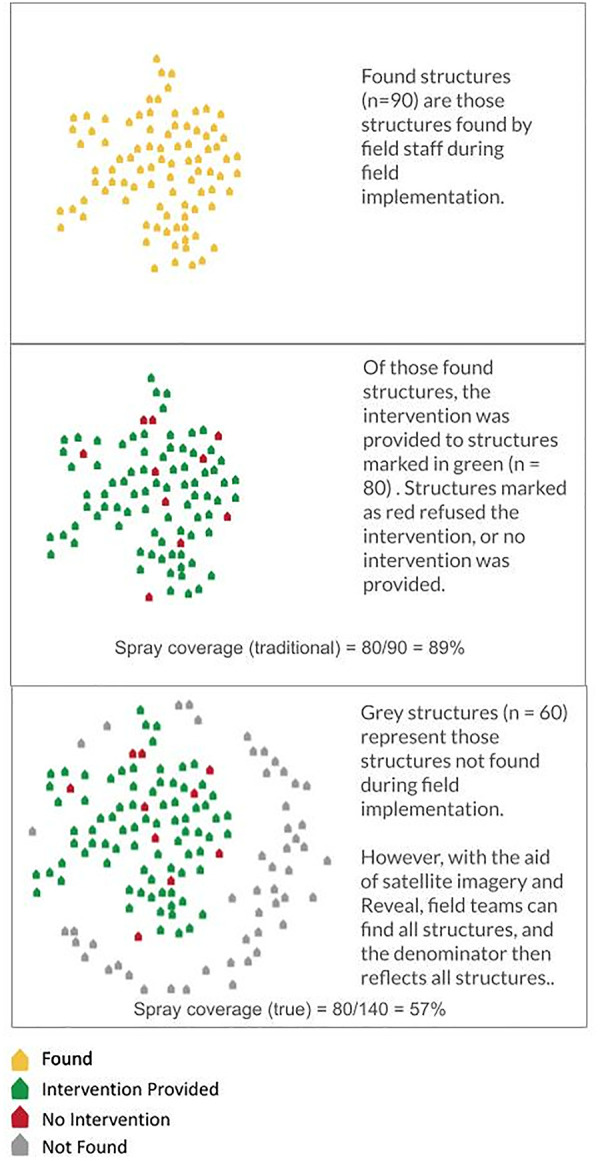
Structures may go undiscovered, and thus not be included in the denominator of spray coverage calculations. Falsely low denominators result in spray coverages being overreported.

Several studies evaluating achieved IRS coverage suggest that teams conducting spray operations may underestimate total structures within targeted areas due to inaccurate or unavailable structure or population information [8–11]. In 2014 in two districts in Zambia, paper maps demarcating the location of each structure were provided to spray teams as they conducted IRS, while in a third district, no such maps were provided [8]. Research surveyors later visited randomly selected transects of structures in each district and discovered that the percentage of all structures sprayed was significantly lower (54% versus 65 and 69%) in the district without maps compared to the two with maps [8]. However, the reported programmatic coverages of these districts were all over 90% [8]. IRS campaigns in Iran, Namibia, and South Africa also report frequently missing structures, sometimes when they are geographically hard to reach, but at times even when their community is reached, but their individual house is not found [9–11]. A basic problem in clearly defining and reporting coverage is that such “not found” structures are often excluded from the coverage denominator (**Fig 1**). As in Zambia in 2014, excluding these structures leads to overestimated coverage reports [12]. Overestimated coverage can impact IRS efficacy: the WHO recommendation for an 80% coverage threshold comes from studies that saw greater declines in malaria prevalence and incidence when IRS coverage was higher at sub-district and community levels [5,13,14].

Historically, coverage calculations relied on data collected through standardized paper data collection forms; spray operators collected a line of data on each household visited, which was aggregated to an administrative unit (e.g., district, province) to calculate coverage at that level. Many countries now store data in electronic databases, and several use electronic methods for data collection. Between 2012 and 2014, two independent digital data collection tools for IRS, both called ‘mSpray,’ emerged in pilot testing; one in Limpopo, South Africa, and the other in northern Zambia [8,15]. The latter of the two, now termed “Reveal”, has been further developed and broadly adopted, and is the subject of this work. Between 2017 and 2021, malaria programs in Namibia, Botswana, and Madhya Pradesh, India deployed similarly digitized IRS tracking tools [16,17]. The University of Oslo, developer of the widely used health management information system database, DHIS2, supports a vector control data collection module, which is currently in use in dozens of countries [18]. Several of these tools collect household structure GPS locations, providing spatial measurements of spray coverage, and enabling real-time use of data in management of operations, such as reviewing daily progress to flag missed households or communities for sensitization or revisit [8,15]. This immediacy of response is an improvement from paper-based collection systems that require manual data entry, which causes lags in data review and decision making [8,15,17].

Here, we describe the methodology that the Reveal tool follows for generating structure counts and spray coverage measures to guide IRS campaigns. We also present structure and coverage estimates of the Zambia National Malaria Elimination Programme’s (NMEP) Reveal implementation from 2016 to 2020. Lastly, we utilize the Reveal structure-level dataset generated from IRS spray campaigns in these years to examine patterns in campaign outcomes associated with different implementation conditions.

## Materials and methods

### Implementation

**Fig 2:**
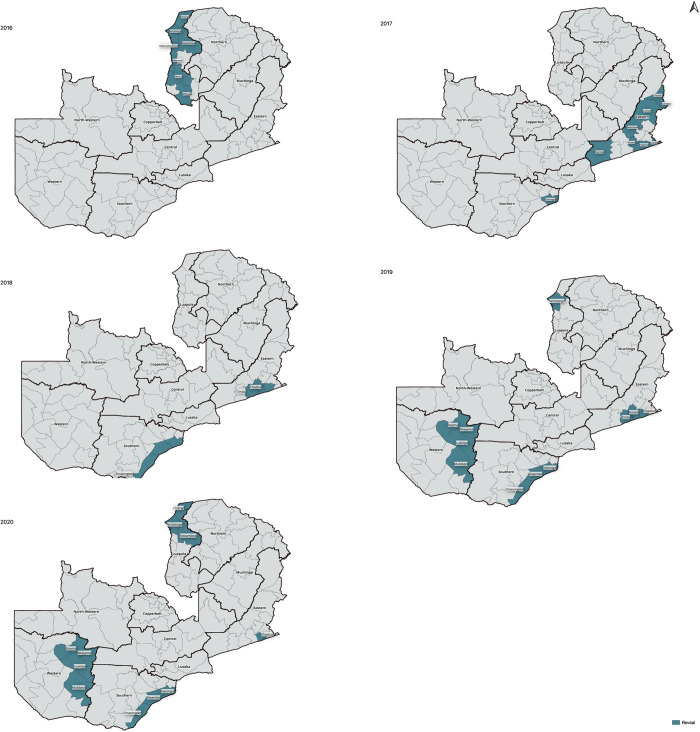
Locations and timing of Reveal implementation in Zambia between 2016 and 2020. Boundary shapefiles were obtained through the Humanitarian Data Exchange under a Creative Commons Attribution 4.0 International License. https://creativecommons.org/licenses/by/4.0/legalcode.

Twenty-one districts in four Zambian provinces implemented Reveal between 2016 and 2020 (**Fig 2**). Funding support was provided by PMI through the Africa Indoor Residual Spraying Project II and the VectorLink Project, and the Bill and Melinda Gates Foundation (BMGF) through the Malaria Control and Elimination Partnership in Africa (MACEPA). Several districts implemented Reveal for multiple years, such that there were a total of 40 district-years of implementations (see Fig 2), but not all implementation years were consecutive due to changing funding availability and priorities. Ten districts implemented Reveal for one year, five districts for two years, four districts for three years, and two districts, Chadiza and Siavonga, implemented for four years.

### Structure and coverage estimations

**Fig 3:**
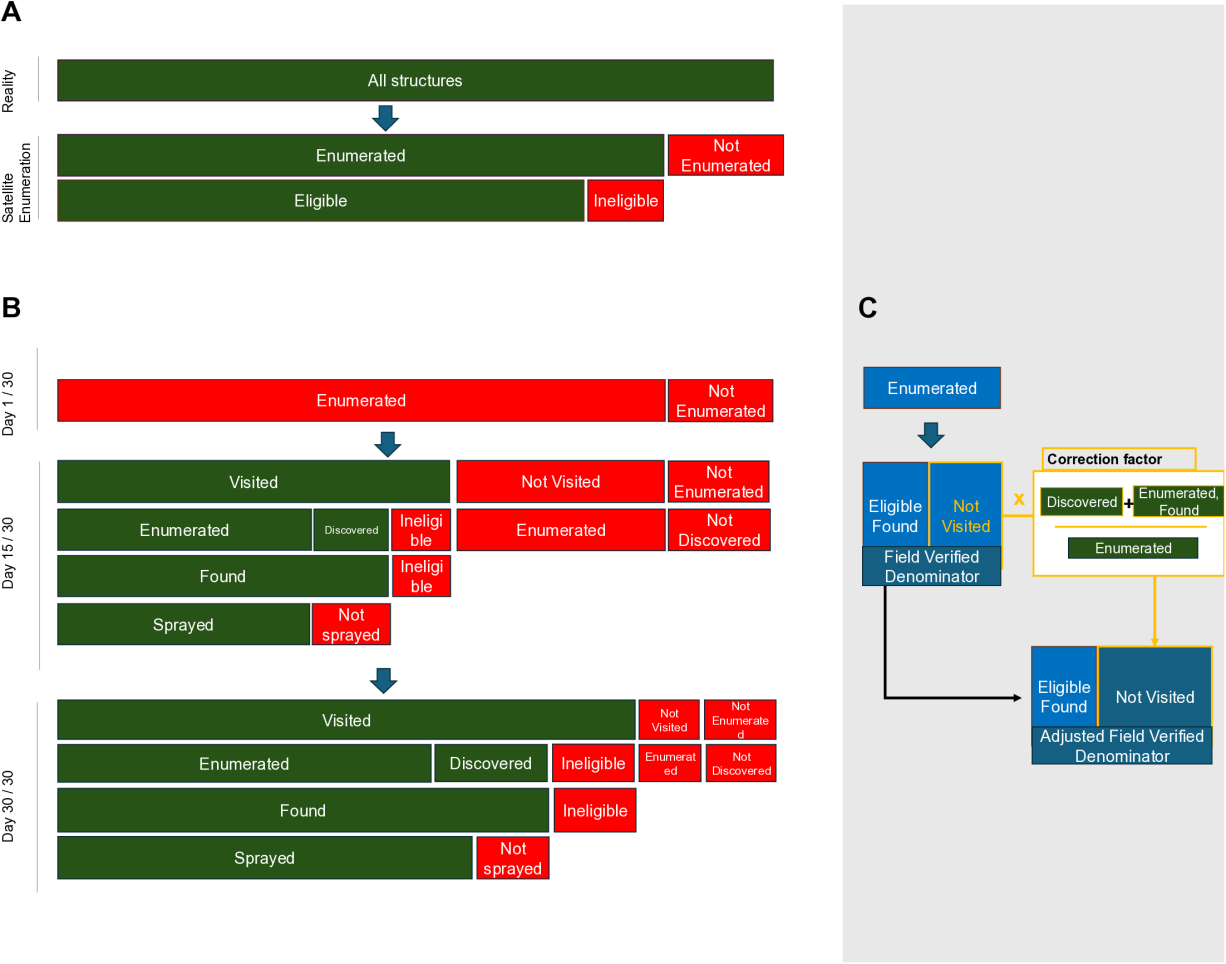
The relationship between structure counts as defined in Table 1 is provided in a tree format in different stages of implementation. Total structures included both those structures that are initially enumerated and those structure that were added during the field enumeration. In the enumeration stage (A) structures were either enumerated or not enumerated, and though this is unknown, some of the enumerated structures were ineligible. When the spray campaign starts (B, Day 1) no structures are sprayed. By Day 15, some structures are visited: some are eligible and sprayed, some are ineligible, and additional unenumerated eligible structures will be discovered and sprayed. Some structures are not visited, which includes both some enumerated and not enumerated structures. By Day 30, more structures will be visited, determined eligible/ineligible, discovered, and sprayed if eligible. A proportion of structures remain that are enumerated and not visited and not enumerated and not discovered. The adjusted field verified denominator is calculated by adjusting the not visited count by the ratio of discovering structures and determine structures are ineligible, compared to all structures that were enumerated and visited.

Two stages of the approach of the Reveal tool contribute to structure estimates: A) enumeration and B) field visits, during which structures are assigned various statuses that inform their inclusion in the numerator and denominators used to calculate coverage. Structure status and spray coverage definitions are described below and summarized in tabular and visual formats in **Table 1** and **Fig 3**.

**Table 1:** Structure counts and spray coverage calculations. * indicators that are collected in traditional spray programs. † indicators that are collected in spray programs using Reveal or similar approach.

Indicator Name	Calculation	Definition
**Structure status from enumeration of satellite imagery**		
Enumerated†	–	Suspected residential structures identified using satellite-based imagery. Enumerators are trained to identify features of structures that are more often residential (i.e., shape, size, proximity to road).
**Structure status during field visits**		
Visited†	–	Structures visited during implementation with data collected against them. This is independent of whether the structure was enumerated and independent of whether considered eligible for IRS.
Discovered†	–	Eligible residential structures that were not enumerated but discovered during implementation.
“Found” (Eligible)*†	–	Visited structures confirmed eligible (includes enumerated and confirmed eligible and discovered structures).
Ineligible†	–	Visited structures confirmed ineligible.
Sprayed*†	–	Eligible, visited structures, recorded with a status of “sprayed”.
Not Sprayed*†	–	Eligible, visited structures, recorded with a status of “not sprayed”.
Not Visited†	–	Enumerated structures that were not visited in the campaign; these are assumed to be eligible.
**Denominator Calculations**		
Field-Verified-Denominator†	Found + Not Visited	Structures that were found (visited and confirmed eligible) irrespective of enumeration status AND the number of enumerated structures not visited (without eligibility confirmed).
Adjusted Field-Verified-Denominator†	Found + [Not Visited * (Discovered – Ineligible)/ Visited]	Field-verified-denominator, adjusted based on the proportion of enumerated structures that could not be confirmed eligible.
**Coverage calculations**		
Spray Coverage (Traditional)*†	Sprayed/ Found	The proportion of eligible structures found during spray operations that were sprayed. This was used in traditional programmatic measurement where a satellite enumeration estimate was not available and there was no quantification of “Not Visited” structures. This will overestimate true spray coverage if “Not Visited” is high.
Spray Coverage (True)†	Sprayed/ Field-Verified-Denominator	The proportion of structures in the field verified denominator that were sprayed.
Found Coverage†	Found/ Field-Verified- Denominator	The proportion of eligible structures on-the-ground that were found during spray operations.
Adjusted Spray Coverage (True)†	Sprayed/ Adj. Field-Verified- Denominator	The proportion of eligible structures on-the-ground that were sprayed, adjusted based on the proportion of enumerated that could not be confirmed eligible.
Adjusted Found Coverage†	Found/ Adj. Field-Verified- Denominator	The proportion of eligible structures on-the-ground that were found during spray operations, adjusted based on the proportion of suspected eligible structures that could not be confirmed eligible.

### Structure status

#### A. Enumeration.

Teams of trained enumerators digitally map areas of interest through visual review of satellite imagery in GIS software. They use contextual knowledge to identify and manually capture the GPS locations of suspected residential structures which are then considered “Enumerated” (Table 1, Fig 3). A residential structure is a permanent construction that people identify as their residence or sleeping space [19]. In 2016, spray efforts were directed at densely populated parts of districts, so enumeration followed a focused approach: first, enumerators panned through a grid of one-square kilometer satellite imagery tiles and flagged tiles with at least 10 structures clustered closely together that appeared to be a village or large household complex. Second, they returned to each marked tile and individually enumerated each structure on that tile. In 2017 – 2020, a complete enumeration approach was used, where enumerators reviewed each tile one-by-one, individually enumerating each structure across all tiles. In districts using the Reveal tool for a second, third, or fourth year, the complete enumeration process was used, but the enumerators reviewed satellite imagery overlaid with the previous year’s enumerated and field-updated structures. When enumeration of a district was complete, “spray areas” were created. Spray areas were clusters of spatially proximal structures divided by natural environmental boundaries (e.g., a road or river) and operational size limitations (such that a spray area exceeding a certain structure count is subdivided). These spray areas acted as operational units to which teams could be deployed for daily spray activities and against which IRS coverage was reported. Details of the enumeration and spray area creation methodology are described elsewhere [20].

#### B. Field visits.

In a series of microplanning meetings, spray campaign planners determined which spray areas would be sprayed using the Reveal tool web-based planning module, which provided an interface for each district showing maps of spray areas and enumerated structures within each, and allowing planners to “select” which would be targeted for spray. Then, during implementation, the Reveal tool mobile application aided on-the-ground teams to navigate to selected spray areas and individual structures. This application was Android-based and showed users a map demarcated with structures in the area they were working within. The map included a navigational feature that showed users where they were as they moved around the areas, and it allowed them to tap on a structure when nearby to capture information. At each structure, a spray operator captured information against the structure, at which point it was considered “Visited”. Visited structures may be 1) pre-enumerated structures that were verified as eligible, 2) pre-enumerated structures that were visited and determined “Ineligible”, and 3) structures that were missed in the enumeration process but “Discovered” during field visits and determined eligible (**Table 1**, **Fig 3**). In Zambia, a structure is “eligible” if it is a residential structure. We defined “Found” structures as those visited and confirmed eligible, regardless of whether they were initially enumerated or discovered; this is the standard terminology used by the malaria program and the denominator used in traditional spray coverage calculation (see Coverage calculations) (**Table 1**, **Fig 3**). Spray operators documented whether each visited, eligible structure was “Sprayed” or “Not sprayed.” They also captured basic demographic information on structure residents and net ownership.

### Denominators

Ineligible and discovered structures were respectively subtracted and added to the enumeration estimate of structure count to yield a “field-verified-denominator” estimate (**Table 1**, **Fig 3**) which is equivalent to the sum of found and not visited structures. This was considered the best estimate of total eligible structure count and served as the denominator for true coverage calculations. The count of not visited structures in the field-verified-denominator were then adjusted by a correction factor (defined as the ratio of the number of eligible structures visited to the number of visited structures that had been enumerated). This accounted for structures that would be likely be added and removed if these structures were all visited.

### Coverage calculations

We divided sprayed structures by found structures to calculate the “Spray Coverage (Traditional)” as it has been traditionally estimated by spray programs; these are the indicators typically included in programmatic reports and evaluations of IRS. To estimate “Found Coverage”, confirmed eligible were divided by the field-verified-denominator structures to estimate. For “Spray Coverage (True)” sprayed structures are divided by the field-verified-denominator. For each coverage measurement, we presented an unadjusted and adjusted estimate, where the later uses the “Adjusted-Field-Verified-Denominator” (**Table 1**, **Fig 3**).

During the IRS campaign, structure estimates and coverage calculations were calculated at the level of the spray areas and presented on real-time Reveal tool web-based dashboards. These allowed campaign managers to monitor daily progress and direct teams to revisit areas if coverage was low. For this analysis, we focused on structure counts and coverage in each district for each year (the district-year) of Reveal implementation. We also calculated the difference in “Spray Coverage (Traditional)” and “Adjusted Spray Coverage (True)”.

### Implementation conditions

The enumeration approach, IRS targeting strategies, partner support, and operational goals varied in each district-year. The enumeration approach was focused or complete, as described in the enumeration methods, above. The IRS targeting strategy was either “universal,” in which the program aims to spray 80% or more of all structures in a district, or “targeted”, which selected a subset of structures within a district [21]. Universal targeting is also known as “blanket coverage.” The type of partner support between the malaria program and external partners and funders also varied. In some district-years, partners provided only technical assistance, whereas in others they also provided all funding (“Implementation resourcing,” **S1 Table**) and managed the implementation of the campaigns (“In-field oversight,” **S1 Table**). Technical assistance included funding microplanning meetings prior to IRS campaigns and Reveal support during the campaign. Reveal support included application software setup for that district-year, provision of mobile tablets for data collection, hiring of additional spray team leaders responsible for navigating to and documenting against spray areas and structures, and hiring of a Reveal surveillance officer for each district. A summary of these strategies across district-years and the details of each district-year are in **Table 2** and **S1 Table** respectively.

**Table 2:** Implementation conditions summarized across district-years.

	Number of district-years
**Partner support**	
Technical assistance, implementation resourcing, in-field oversight	23
Technical assistance, implementation resourcing	10
Technical assistance	7
**Enumeration strategy**	
Enumerate clusters of 10 + structures	7
Enumerate all	33
**Targeting strategy**	
Targeted	31
Universal	9

### Found and spray coverage patterns

Given the highly variable combinations of implementation conditions across district-years, multivariable analysis was necessary to examine their association with structure-level spray campaign outcomes. We built two logistic regression models using structure-level data to explore differences in two outcomes: 1) the odds of a structure being sprayed, and 2) the odds of a structure being found. We hypothesized that the following factors might affect these odds: the campaign calendar year, targeting strategy (universal or targeted), partner support level (technical assistance only or technical assistance with implementation resourcing and/or in-field oversight), and the year of Reveal implementation in a given district (first, second, third, or fourth). We conducted all data cleaning, descriptive analyses, and regression analysis using Stata version 15 [22]. Figures were constructed in R (version 4.2.3) [23].

## Results

### Structure and coverage estimates

A total of 1,304,708 structure records were in the Reveal-structure-level dataset compiled from 2016 -2020 structure estimates. Of these, 69% were visited: 50% sprayed, 5% not sprayed (though eligible), 14% ineligible, and 31% were not visited. 1,124,716 structures were eligible for spray after removing structures marked as ineligible (175,524 [13%] total over all district-years) and adding discovered structures (157,601 [12%] total over all district-years); this includes not visited structures still assumed to be eligible. Of these, 713,135 (63%) were marked as found and 646,980 (58%) were marked as sprayed during the spray campaigns.

**Fig 4:**
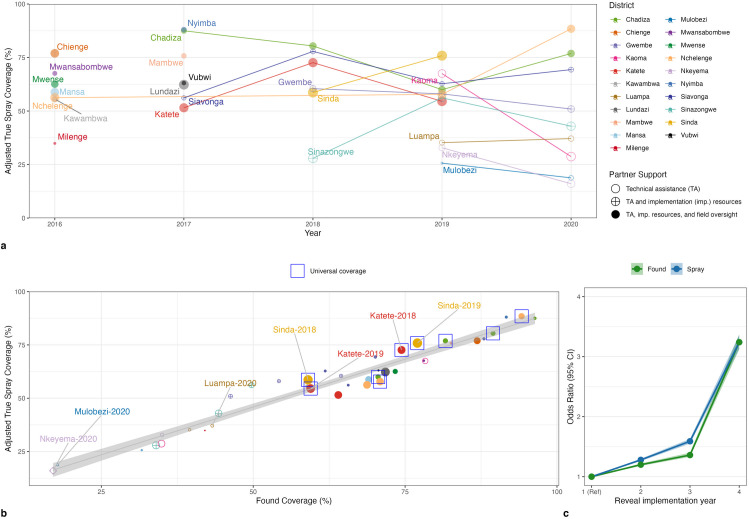
Panel A. True spray coverage by district and year is shown. Points are proportional to the adjusted field eligible denominator in a given year. Panel B. True spray coverage versus found coverage is shown. High found and high spray coverage district-years Nchelenge 2020, and Chadiza 2018 were years where the targeting strategy was universal coverage. Panel C. Odds ratios for continued years of Reveal implementation within districts, derived from multivariate analyses in Table 3.

The mean adjusted field-verified-denominator structure count in each district-year was 27,727 with a range in the size of districts from 5,826–55,997 structures (**S2 Table**). The initial enumeration process underestimated the adjusted structure count in 22 of 40 district-years and overestimated the adjusted structure count in 18 district-years. In districts that implemented Reveal for more than one year, enumeration sensitivity increased by 13 percentage points from the first to the second year. Mean found coverage was 62.7% ranging from 17.0 to 96.4%. The district-years with the highest found coverage were Chadiza-2017 (96%), Nchelenge-2020 (94%), and Nyimba-2017 (92%), notably district-years with high levels of technical assistance and targeted for universal coverage. The mean adjusted true spray coverage across all district-years was 57%. Thirty-one district-years reported district spray coverage above 85%, whereas only three district-years had a true district-wide adjusted coverage above 85%. The district-years with the highest adjusted true spray coverage were Chadiza-2017 (88%), Chadiza-2018 (80%), Nyimba-2017 (88%), and Nchelenge-2020 (88%). All programmatic reported coverages overestimated the true adjusted coverages, with a mean overestimate of 31.5 percentage points and a range of 2.8 and 69.4. Within district, year-to-year trends were not consistent across districts, likely because they were driven by changing implementation scenarios. Several districts (Katete, Sinda, Siavonga, Sinazongwe) saw notable increases in spray coverage in their second, relative to their first year of Reveal implementation (**Fig 4, Panel A**). However, spray coverage in the second year of Reveal use in Chadiza District, and most districts in Western Province, most notably in Kaoma District, decreased relative to the first year. Chadiza’s second year of Reveal implementation expanded to rural areas where there were higher refusals. Western Province’s second year of Reveal implementation was 2020, when financial resources were redirected from IRS to COVID-19 response. High found and high spray coverage district-years Nchelenge 2020, and Chadiza 2018 were years where the targeting strategy was universal coverage (**Fig 4, Panel B**). Nyimba and Chadiza 2017 were also high performing though not conducting universal targeting; these smaller districts are in Eastern Province, where there was an especially well-resourced campaign in 2017. District-years with high found but low spray coverage (e.g., Chiengi 2016 and Siavonga 2018) indicate teams planned well to reach all areas, but during the IRS campaign did not spray them at high coverage, due to poor sensitization and/or refusals.

Low found coverage is expected when using a non-universal, targeting strategy as in 2019 and 2020 in Luampa, Mulobezi, Nkeyema Districts in Western Province and in Katete District in 2019 (in contrast to Katete District in 2018, which had higher found coverage under a universal coverage strategy). Low found coverage is not expected in district-years aiming for universal coverage and may be reflective of challenges in planning. For example, Sinda District, a district newly created in 2012, was targeted for universal coverage in its first year of spray in 2018. However, microplanning and chemical procurement took place before satellite imagery-based enumeration was complete and instead relied on headcount and census estimates. As a result, the total structure count was underestimated, and found (59%) and spray coverage (57%) were low. When Sinda District used corrected enumeration estimates in the 2019 campaign, found (77%) and spray (75%) coverage improved.

### Exploratory analysis

In multivariable analysis, where other implementation conditions are held constant, the household odds of being sprayed and the odds of being found were both highest in 2017 and lowest in 2020. In 2017, household odds of spray were 13% higher than in 2016 (Odds Ratio [OR]: 1.13, 95% Confidence Interval [CI]: 1.12 – 1.15, Table 3) and odds of being found were 23% higher (OR 1.23, 95% CI: 1.21 – 1.24). In 2020, household odds of spray were 66% lower than in 2016 (OR 0.34, 95% CI: 0.34 – 0.35, Table 3) and odds of being found were 53% lower (OR 0.47, 95% CI: 0.47 – 0.48). In 2017, Reveal supported spray in 6 districts in Eastern Province, where there was an operational research study that called extra attention to planning and spray efforts, in addition to the spray itself being well-resourced [24]. In 2020, Reveal supported spray in 9 districts, the majority of which had a narrower targeting strategy. In district-years where a universal coverage strategy was deployed, the household odds of being sprayed was, logically, 72% higher (OR: 1.72, 95% CI: 1.68 – 1.75) and the odds of being found was 24% higher (OR: 1.24, 95% CI: 1.21 – 1.26). Most strikingly, the odds of finding and spraying structures increases with year of Reveal implementation; by the fourth year of implementation in an average district, household odds of being sprayed and found were both over 2 times higher than the first year of implementation (**Fig 4, Panel B**, sprayed OR: 3.24, 95% CI: 3.13 – 3.36, found OR: 3.24, 95% CI: 3.12 – 3.37).

**Table 3:** Two multivariable logistic regressions were run to examine associations between implementations conditions and the odds of a household being sprayed or found.

	Spray OR			Found OR		
		95% CI	p-value		95% CI	p-value
Targeting Strategy						
Targeted, non-universal	Ref.		.	Ref.		.
Universal coverage	1.72	1.68,1.75	<0.01	1.24	1.21,1.26	<0.01
Partner Support						
Technical assistance (TA)	Ref.		.	Ref.		.
TA and Implementation resources	1.41	1.39,1.44	<0.01	1.35	1.33,1.38	<0.01
TA, Implementation resources, and field oversight	1.83	1.81,1.84	<0.01	2.55	2.52,2.57	<0.01
Calendar Year						
2016	Ref.		.	Ref.		.
2017	1.13	1.12,1.15	<0.01	1.23	1.21,1.24	<0.01
2018	0.61	0.59,0.62	<0.01	0.73	0.72,0.74	<0.01
2019	0.51	0.50,0.51	<0.01	0.72	0.71,0.73	<0.01
2020	0.34	0.34,0.35	<0.01	0.47	0.47,0.48	<0.01
Reveal Implementation						
Year 1	Ref.		.	Ref.		.
Year 2	1.28	1.26,1.30	<0.01	1.20	1.18,1.22	<0.01
Year 3	1.59	1.55,1.63	<0.01	1.36	1.33,1.40	<0.01
Year 4	3.24	3.13,3.36	<0.01	3.24	3.12,3.37	<0.01

OR = odds ratio, TA = technical assistance.

Throughout the implementation scenarios, the Reveal tool mobile application and web-based dashboard experienced a range of in-field challenges – these were related to significant architectural updates made to the platform, challenges to providing geospatial data for planning and navigation in areas of low connectivity, and necessary user experience and user interface improvements. Although teams were hampered during several of the deployment scenarios due to technical issues, overall the platform’s up-time, syncing challenges and user experience improved over the course of the project.

## Discussion

The success of spray campaigns in reaching and spraying structures is in large part dependent on the resources available to conduct the campaign: we found that where universal coverage was planned, where more implementation support was provided, and where the Reveal tool was implemented across several years, there were higher odds of finding and spraying structures. The enumeration process both over and under-estimated total structure counts across districts, but enumeration accuracy improved as districts implemented Reveal over consecutive years. The improvement in enumeration accuracy likely influenced the reported increases in coverage in subsequent years of Reveal implementation, by allowing spray teams to be more efficient, spending less time going to spray areas that were ultimately ineligible. The increases in coverage across years of implementation likely also reflect iterative improvement to the technology of the tool itself and the districts staff’s use of it. Like any electronic reporting system, implementation requires technical support to manage hardware and software, and can be hampered by infrastructural challenges including network connectivity and access to power.

The coverage indicators captured from 2016-2020 in Zambia highlight several problems with the measurement metrics traditionally used to evaluate and communicate results of IRS campaigns. First, programmatic reports imply that traditional spray programs deliver services more broadly than they actually do. Reports will list a given district as having received IRS, when only a portion of residents of that district will receive IRS; only 62% of eligible structures across all district-years were found by the spray program. Amongst districts with planned universal coverage, still only 73% of structures were found. While this represents substantial investment, it reminds us that district-level IRS implementations are rarely district-wide, and programmatic reports do not always represent a district-wide view of service delivery. Second, although traditionally reported spray coverage captures whether or not teams spray houses once they reach them, this metric consistently and greatly overestimates coverage, by an average of 30 percentage points. This communications gap fails to raise the alarm of potentially ineffective IRS implementation -- in **S2 Table** we presented several examples of district-years where reported spray coverage was above 90% but true coverage was below 80%, and in several cases was below 60%. Only three of 31 districts reporting coverage above the programmatic threshold of 85%, had true coverage above 85%. Inaccurate estimates of IRS implementation will also influence perception of IRS effectiveness. An evaluation may look at districts that have reportedly received universal coverage and determine IRS has not had as large an impact.

There are important limitations to this work in relating coverage to actual impact. First, we exclusively discuss indicators of IRS coverage, but IRS is not the only vector control intervention. The Zambia National Malaria Elimination Strategic Plan takes a “mosaic” approach to vector control, where areas that are not targeted by IRS are included in tri-annual distributions of long lasting insecticide treated nets (LLINs) [25]. Therefore, while this data highlights the gap between programmatic coverage and actual population-based coverage, we cannot assume that this gap necessarily represents unprotected households. While we do not report on LLIN coverage in this analysis, that data is collected at each structure, and analyses of LLIN coverage using this data, or in other applications of Reveal, are possible. Further, Reveal measured coverage at the structure level per Zambia program definitions, where other countries utilize room-level coverage estimates that add a layer of granularity helpful to impact evaluations. Lastly, we summarize coverage measurements at the district level, while analyses of IRS coverage in Zambia suggest that high coverage at finer spatial scales is associated with reduced odds of malaria infection [26]. For these reasons we did not attempt to link IRS coverage to disease burden, choosing instead to focus on the methodologies of generating these indicators. While impact evaluation was out of scope for this work, it could be further explored in the future – this is a household level dataset and observations included LLINs counted in each house. That said, a separate study of Nchelenge District, Zambia in 2017 found that when Reveal and its metrics were used in IRS campaigns, declines in malaria incidence were larger than in other districts implementing IRS without Reveal [27]. Similar geospatial approaches have also demonstrated impact. In Sri Lanka, sub-district level mapping of malaria risk allowed focused control and elimination efforts in response to epidemic forecasting and contributed to the significant progress made towards elimination [28]. Polio vaccination campaigns in Nigeria managed a similar process of community-level interventions by relying on detailed understanding of the location of human settlements to support “microplanning”, or the assignment of specific settlements to specific teams [29].

To increase IRS campaign coverage requires greater transparency into measured coverage and improved tools, but enabling this transparency requires additional resources be available for already resource-intensive IRS campaigns. The Reveal tool, while open-source, has one-time development and deployment and recurrent operational costs, making it a non-insubstantial investment, that becomes cost effective at scale and over time. A costing study estimated a single, one-time district deployment of Reveal costs an $156,563 (11% of a single-district IRS budget of $1,337,359), but when scaled to a full province of 11 districts over 5-years costs $89438 across 11 districts (1% of full province IRS budget of $6,686,795) [30]. The costing study, which used data from the 2017 Nchelenge District (one of the districts in this analysis), found a 21% reduction in cost per case averted when using Reveal with IRS compared to IRS alone [27,30]. [Cost per case averted with IRS alone was estimated at $18.81 compared to cost per case averted when the geospatial tooling was added ($16.01 in year one, dropping to $14.42 by year five)] [31]. Still, resources may not always be available to support a digital-mobile-phone based data collection alongside IRS, and simplified, less resource-intensive approaches have conducted imagery-based enumeration structure estimation for campaign planning, and limited mobile data collection and monitoring to the community (rather than household)-level. More recently, automated datasets derived from satellite imagery have provided structure estimates for planning when manual digital enumeration was not possible; in 2021, the Zambia NMEP used building footprints with Reveal-adjusted residential structure counts to plan for complimentary IRS and ITN campaigns across the country [32].

This analysis described a geo-enabled approach to measurement of IRS deployment in Zambia and provided an initial exploration of implementation factors and their associations with IRS coverage. In summary, the approach 1) identified more accurate, but significantly lower estimates of true adjusted spray coverage than programmatic spray coverage metrics (by an average of 31.5 percentage points), 2) improved spray and found coverage in each successive year of implementation and 3) reveled that universal coverage and increased partner support improved found and spray coverage. Reveal indicators provided a better characterization of the progress of spray activities and the proactive use of this data throughout the spray campaign likely improved spray coverage and implementation effectiveness.

## Supporting information

S1 Table Between 2016 and 2020, there were 40 implementations of Reveal across numerous districts. Each district implementation was carried out by the National Malaria Elimination Programme, with funding and implementation support from different entities and of different kinds. In 2016, the first year of Reveal deployment, enumerators only enumerated structures if they were a part of a cluster of at least 10 structures. The targeting in a district in a given year was determined by the amount of resources (insecticide volume and human resources) available in that district.
(DOCX)

S2 Table Structure estimates and spray coverage estimate are presented by district-year. (DOCX)
