# Supplementary material for: A geo-enabled digital tool for microplanning and delivery of indoor residual spray in Zambia: A case study, 2016–2020
Source: PLOS Glob Public Health. 2025 Nov 20;5(11):e0004683. doi: 10.1371/journal.pgph.0004683 (PMC12633927; doi:10.1371/journal.pgph.0004683)
Supplement: S1 Table — Each district implementation was carried out by the National Malaria Elimination Programme, with funding and implementation support from different entities and of different kinds. In 2016, the first year of Reveal deployment, enumerators only enumerated structures if they were a part of a cluster of at least 10 structures. The targeting in a district in a given year was determined by the amount of resources (insecticide volume and human resources) available in that district. (DOCX) [file pgph.0004683.s001.docx]

S1 Table. Between 2016 and 2020, there were 40 implementations of Reveal across numerous districts. Each district implementation was carried out by the National Malaria Elimination Programme, with funding and implementation support from different entities and of different kinds. In 2016, the first year of Reveal deployment, enumerators only enumerated structures if they were a part of a cluster of at least 10 structures. The targeting in a district in a given year was determined by the amount of resources (insecticide volume and human resources) available in that district.

| **Year** | **District** | **Funding support** | **Implementation support** | **Type of support** | **Enumeration strategy** | **Targeting strategy** |
| --- | --- | --- | --- | --- | --- | --- |
| 2016 | Chienge | PMI | AIRSII | Technical assistance, implementation resourcing, in-field oversight | Enumerate clusters of 10+ structures | Targeted |
| 2016 | Kawambwa | PMI | AIRSII | Technical assistance, implementation resourcing, in-field oversight | Enumerate clusters of 10+ structures | Targeted |
| 2016 | Mansa | PMI | AIRSII | Technical assistance, implementation resourcing, in-field oversight | Enumerate clusters of 10+ structures | Targeted |
| 2016 | Milenge | PMI | AIRSII | Technical assistance, implementation resourcing, in-field oversight | Enumerate clusters of 10+ structures | Targeted |
| 2016 | Mwansabombwe | PMI | AIRSII | Technical assistance, implementation resourcing, in-field oversight | Enumerate clusters of 10+ structures | Targeted |
| 2016 | Mwense | PMI | AIRSII | Technical assistance, implementation resourcing, in-field oversight | Enumerate clusters of 10+ structures | Targeted |
| 2016 | Nchelenge | PMI | AIRSII | Technical assistance, implementation resourcing, in-field oversight | Enumerate clusters of 10+ structures | Targeted |
| 2017 | Chadiza | PMI | AIRSII | Technical assistance, implementation resourcing, in-field oversight | Enumerate all | Targeted |
| 2017 | Katete | PMI | AIRSII | Technical assistance, implementation resourcing, in-field oversight | Enumerate all | Targeted |
| 2017 | Lundazi | PMI | AIRSII | Technical assistance, implementation resourcing, in-field oversight | Enumerate all | Targeted |
| 2017 | Mambwe | PMI | AIRSII | Technical assistance, implementation resourcing, in-field oversight | Enumerate all | Targeted |
| 2017 | Nyimba | PMI | AIRSII | Technical assistance, implementation resourcing, in-field oversight | Enumerate all | Targeted |
| 2017 | Siavonga | PMI | AIRSII | Technical assistance, implementation resourcing, in-field oversight | Enumerate all | Targeted |
| 2017 | Vubwi | PMI | AIRSII | Technical assistance, implementation resourcing, in-field oversight | Enumerate all | Targeted |
| 2018 | Chadiza | PMI | AIRSII | Technical assistance, implementation resourcing, in-field oversight | Enumerate all | Universal |
| 2018 | Gwembe | BMGF | MACEPA | Technical assistance, implementation resourcing | Enumerate all | Targeted |
| 2018 | Katete | PMI | AIRSII | Technical assistance, implementation resourcing, in-field oversight | Enumerate all | Universal |
| 2018 | Siavonga | BMGF | MACEPA | Technical assistance, implementation resourcing | Enumerate all | Targeted |
| 2018 | Sinazongwe | BMGF | MACEPA | Technical assistance, implementation resourcing | Enumerate all | Targeted |
| 2018 | Sinda | PMI | AIRSII | Technical assistance, implementation resourcing, in-field oversight | Enumerate all | Universal |
| 2019 | Chadiza | PMI | VectorLink | Technical assistance, implementation resourcing, in-field oversight | Enumerate all | Universal |
| 2019 | Gwembe | BMGF | MACEPA | Technical assistance, implementation resourcing | Enumerate all | Targeted |
| 2019 | Kaoma | BMGF | MACEPA | Technical assistance, implementation resourcing | Enumerate all | Targeted |
| 2019 | Katete | PMI | VectorLink | Technical assistance, implementation resourcing, in-field oversight | Enumerate all | Universal |
| 2019 | Luampa | BMGF | MACEPA | Technical support | Enumerate all | Targeted |
| 2019 | Mulobezi | BMGF | MACEPA | Technical support | Enumerate all | Targeted |
| 2019 | Nchelenge | PMI | VectorLink | Technical assistance, implementation resourcing, in-field oversight | Enumerate all | Universal |
| 2019 | Nkeyema | BMGF | MACEPA | Technical support | Enumerate all | Targeted |
| 2019 | Siavonga | BMGF | MACEPA | Technical assistance, implementation resourcing | Enumerate all | Targeted |
| 2019 | Sinazongwe | BMGF | MACEPA | Technical assistance, implementation resourcing | Enumerate all | Targeted |
| 2019 | Sinda | PMI | VectorLink | Technical assistance, implementation resourcing, in-field oversight | Enumerate all | Universal |
| 2020 | Chadiza | PMI | VectorLink Project | Technical assistance, implementation resourcing, in-field oversight | Enumerate all | Universal |
| 2020 | Gwembe | BMGF | MACEPA | Technical assistance, implementation resourcing | Enumerate all | Targeted |
| 2020 | Kaoma | BMGF | MACEPA | Technical support | Enumerate all | Targeted |
| 2020 | Luampa | BMGF | MACEPA | Technical support | Enumerate all | Targeted |
| 2020 | Mulobezi | BMGF | MACEPA | Technical support | Enumerate all | Targeted |
| 2020 | Nchelenge | PMI | VectorLink | Technical assistance, implementation resourcing, in-field oversight | Enumerate all | Universal |
| 2020 | Nkeyema | BMGF | MACEPA | Technical support | Enumerate all | Targeted |
| 2020 | Siavonga | BMGF | MACEPA | Technical assistance, implementation resourcing | Enumerate all | Targeted |
| 2020 | Sinazongwe | BMGF | MACEPA | Technical assistance, implementation resourcing | Enumerate all | Targeted |

PMI: The President’s Malaria Initiative, BMFG: The Bill and Melinda Gates Foundation, AIRS II: The Africa Indoor Residual Spraying project II, MACEPA: Malaria Control and Evaluation Partnership in Africa
