# Supplementary material for: A geo-enabled digital tool for microplanning and delivery of indoor residual spray in Zambia: A case study, 2016–2020
Source: PLOS Glob Public Health. 2025 Nov 20;5(11):e0004683. doi: 10.1371/journal.pgph.0004683 (PMC12633927; doi:10.1371/journal.pgph.0004683)
Supplement: S2 Table — (DOCX) [file pgph.0004683.s002.docx]

S2 Table. Structure estimates and spray coverage estimate are presented by district-year.

| Year | District | Province | Enumerated | Visited | Field verified eligible | Adjusted field verified eligible | Found coverage (%) | Reported spray coverage (%) | True spray coverage (%) | Adjusted true spray coverage (%) | Reported - true adjusted coverage (%) | Correction factor |
| --- | --- | --- | --- | --- | --- | --- | --- | --- | --- | --- | --- | --- |
| 2016 | Chienge | Luapula | 37506 | 38159 | 39666 | 40014 | 87 | 89 | 78 | 77 | 12 | 1.07 |
| 2016 | Kawambwa | Luapula | 20657 | 13842 | 20055 | 19655 | 59 | 93 | 54 | 56 | 37 | 0.95 |
| 2016 | Mansa | Luapula | 36500 | 28165 | 38605 | 39633 | 69 | 87 | 60 | 59 | 29 | 1.09 |
| 2016 | Milenge | Luapula | 5741 | 3076 | 5980 | 6344 | 42 | 88 | 37 | 35 | 53 | 1.11 |
| 2016 | Mwansabombwe | Luapula | 10977 | 9649 | 11833 | 12098 | 78 | 89 | 69 | 68 | 21 | 1.10 |
| 2016 | Mwense | Luapula | 23729 | 20485 | 27319 | 28907 | 73 | 90 | 66 | 63 | 28 | 1.22 |
| 2016 | Nchelenge | Luapula | 36969 | 31081 | 42191 | 45088 | 69 | 87 | 60 | 56 | 31 | 1.22 |
| 2017 | Chadiza | Eastern | 20822 | 23589 | 17565 | 17462 | 96 | 90 | 87 | 88 | 3 | 0.84 |
| 2017 | Katete | Eastern | 38905 | 32891 | 42779 | 45319 | 64 | 85 | 55 | 52 | 34 | 1.16 |
| 2017 | Lundazi | Eastern | 44996 | 41526 | 50216 | 52618 | 72 | 91 | 65 | 62 | 29 | 1.17 |
| 2017 | Mambwe | Eastern | 13987 | 14844 | 14908 | 15118 | 83 | 93 | 77 | 76 | 17 | 1.08 |
| 2017 | Nyimba | Eastern | 18930 | 20962 | 15948 | 15722 | 92 | 95 | 87 | 88 | 7 | 0.83 |
| 2017 | Vubwi | Eastern | 9315 | 8580 | 10034 | 10367 | 71 | 92 | 65 | 63 | 29 | 1.11 |
| 2017 | Siavonga | Southern | 13455 | 11073 | 11129 | 10205 | 66 | 78 | 51 | 56 | 22 | 0.76 |
| 2018 | Chadiza | Eastern | 20583 | 29020 | 27155 | 28217 | 89 | 93 | 84 | 80 | 13 | 1.37 |
| 2018 | Katete | Eastern | 45769 | 43554 | 46373 | 46584 | 74 | 98 | 73 | 73 | 25 | 1.02 |
| 2018 | Sinda | Eastern | 59644 | 44028 | 56831 | 55029 | 59 | 96 | 57 | 59 | 38 | 0.92 |
| 2018 | Siavonga | Southern | 16260 | 16855 | 12595 | 12219 | 88 | 86 | 76 | 78 | 8 | 0.75 |
| 2018 | Gwembe | Southern | 18193 | 15526 | 18972 | 19431 | 64 | 96 | 62 | 60 | 36 | 1.07 |
| 2018 | Sinazongwe | Southern | 35007 | 14152 | 37062 | 41821 | 34 | 93 | 31 | 28 | 65 | 1.19 |
| 2019 | Chadiza | Eastern | 23573 | 22191 | 27769 | 29995 | 71 | 92 | 65 | 60 | 32 | 1.27 |
| 2019 | Katete | Eastern | 45570 | 35293 | 48638 | 50979 | 59 | 96 | 57 | 55 | 42 | 1.12 |
| 2019 | Sinda | Eastern | 59225 | 58495 | 56708 | 55997 | 77 | 97 | 75 | 76 | 21 | 0.95 |
| 2019 | Siavonga | Southern | 13915 | 9506 | 11611 | 10535 | 62 | 92 | 57 | 63 | 29 | 0.76 |
| 2019 | Gwembe | Southern | 24863 | 15347 | 20832 | 18324 | 54 | 94 | 51 | 58 | 36 | 0.74 |
| 2019 | Sinazongwe | Southern | 45581 | 27192 | 36684 | 30638 | 50 | 94 | 47 | 56 | 38 | 0.67 |
| 2019 | Nchelenge | Luapula | 42869 | 35988 | 44008 | 44494 | 71 | 82 | 58 | 58 | 25 | 1.04 |
| 2019 | Kaoma | Western | 36876 | 29799 | 32608 | 31594 | 78 | 84 | 65 | 68 | 16 | 0.86 |
| 2019 | Luampa | Western | 17809 | 8234 | 15831 | 13531 | 40 | 76 | 30 | 35 | 41 | 0.76 |
| 2019 | Mulobezi | Western | 8608 | 3496 | 7478 | 5826 | 32 | 63 | 20 | 26 | 38 | 0.68 |
| 2019 | Nkeyema | Western | 25542 | 10692 | 22845 | 19099 | 35 | 78 | 27 | 33 | 46 | 0.75 |
| 2020 | Chadiza | Eastern | 29048 | 29999 | 28968 | 28950 | 82 | 94 | 77 | 77 | 17 | 1.00 |
| 2020 | Siavonga | Southern | 18802 | 16481 | 14176 | 12829 | 70 | 90 | 63 | 69 | 20 | 0.68 |
| 2020 | Gwembe | Southern | 27975 | 17066 | 25120 | 22458 | 46 | 98 | 46 | 51 | 47 | 0.80 |
| 2020 | Sinazongwe | Southern | 41754 | 23400 | 40414 | 38848 | 44 | 93 | 41 | 43 | 50 | 0.93 |
| 2020 | Nchelenge | Luapula | 40065 | 46182 | 35896 | 35667 | 94 | 93 | 88 | 88 | 5 | 0.89 |
| 2020 | Kaoma | Western | 39541 | 17887 | 39795 | 40278 | 35 | 83 | 29 | 29 | 55 | 1.02 |
| 2020 | Luampa | Western | 20942 | 12292 | 18122 | 15401 | 43 | 73 | 32 | 37 | 36 | 0.74 |
| 2020 | Mulobezi | Western | 14120 | 3262 | 13526 | 11329 | 18 | 88 | 16 | 19 | 69 | 0.80 |
| 2020 | Nkeyema | Western | 38016 | 9249 | 36471 | 30454 | 17 | 78 | 13 | 16 | 62 | 0.80 |
